# Spheroids from Epithelial and Mesenchymal Cell Phenotypes as Building Blocks in Bioprinting (Review)

**DOI:** 10.17691/stm2025.17.1.11

**Published:** 2025-02-28

**Authors:** D.P. Revokatova, P.I. Koteneva, N.V. Kosheleva, A.I. Shpichka, P.S. Timashev

**Affiliations:** Junior Researcher, Laboratory of Clinical Smart- and Nanotechnologies, Institute of Regenerative Medicine; I.M. Sechenov First Moscow State Medical University (Sechenov University), 8/2 Trubetskaya St., Moscow, 119991, Russia; Junior Researcher, Biofabrika Design Center, Institute of Regenerative Medicine; I.M. Sechenov First Moscow State Medical University (Sechenov University), 8/2 Trubetskaya St., Moscow, 119991, Russia; PhD, Associate Professor, Head of Laboratory of Clinical Smart- and Nanotechnologies, Institute of Regenerative Medicine; I.M. Sechenov First Moscow State Medical University (Sechenov University), 8/2 Trubetskaya St., Moscow, 119991, Russia; PhD, Associate Professor, Head of Laboratory of Applied Microfluidics, Institute of Regenerative Medicine; I.M. Sechenov First Moscow State Medical University (Sechenov University), 8/2 Trubetskaya St., Moscow, 119991, Russia; DSc, Professor, Institute of Regenerative Medicine; Chief Scientific Officer of the Scientific and Technological Park of Biomedicine; I.M. Sechenov First Moscow State Medical University (Sechenov University), 8/2 Trubetskaya St., Moscow, 119991, Russia

**Keywords:** bioprinting, spheroids, tissue engineering, mechanical properties of spheroids, epithelial and mesenchymal cells

## Abstract

Most tissues and organs are based on cells of the epithelial and mesenchymal phenotypes. Epithelial cells build protective barriers, have a key role in absorption and secretion, and participate in metabolism. Characterized by high plasticity and ability to migrate, mesenchymal cells ensure structural support, promote tissue restoration and are important for matrix remodeling. Interaction between these two cell types is critical for maintaining the body integrity and functioning.

Modern tissue engineering is aimed at creation of artificial tissues and organs that have the required cellular composition, mechanical properties and functional potential for medical usage. One of the most popular methods of tissue engineering is 3D bioprinting, which allows creating complex three-dimensional structures with specified characteristics. Recently, special attention has been paid to bioprinting with spheroids being three-dimensional cellular aggregates that can be used as building blocks for tissue-engineered structures. Due to numerous cell-to-cell contacts and accumulation of extracellular matrix, spheroids ensure conditions allowing to form anatomical tissues and organs.

To optimize bioprinting conditions, one shall precisely understand the mechanical properties of spheroids, as they directly affect the ability of cells to migrate and fuse, and thus the rate of construct formation and its overall morphology. This review summarizes the available data on the differences in mechanical properties of epithelial and mesenchymal spheroids, examines methods for their co-culturing in various applications of regenerative medicine, as well as analyzes the peculiarities of their use in different bioprinting methods to obtain high-quality tissue constructs.

## Introduction

In recent decades, tissue engineering has been rapidly developing; in order to solve the problems of regenerative medicine, it combines the achievements of materials science, cell biology, physics, and transplantology. Bioprinting technology is one of the most promising approaches to creation of bioequivalents both for replacement therapy and for preparing adequate models to test the efficacy and safety of personalized medications.

The downward tissue engineering strategy with the formation of three-dimensional constructs followed by injection of a cell suspension thereto is limited by reduced cell viability after transplantation, lack of intercellular contacts and extracellular matrix (ECM). Therefore, the upward approach has recently become popular; it provides bioprinting without cell suspension and uses pre-formed three-dimensional cell aggregates — spheroids, where the cells have formed intercellular contact complexes, synthesized ECM, differentiated in the required direction and adapted to hypoxia [1, 2]. In spheroid-based constructs, viability, proliferation, and functional differentiation potential are significantly higher than in bioprinting based on cell suspension.

Studying the structure and properties of spheroids being a key component of bioinks for tissue creation is a fundamental task of tissue engineering and has an important practical impact. Most tissues and organs are based on cells of epithelial and mesenchymal phenotypes. Epithelial cells form protective barriers, are critical for absorption and secretion, as well as participate in metabolism. Characterized by high plasticity and ability to migrate, mesenchymal cells provide structural support, promote tissue restoration and are important for remodeling. Interaction between these two types of cells is critical for maintaining the body integrity and functionality. Moreover, all processes related to development, morphogenesis, regeneration, and oncogenesis are closely associated with transitions between the epithelial and mesenchymal cell phenotypes, thus the search for new models to study thereof is an important task of regenerative medicine [3]. Combining spheroids from different cell types to form tissue-engineered constructs allows reconstructing complex tissue-specific structures.

This review summarizes the available data on the differences in epithelial and mesenchymal spheroids, examines methods for their co-culturing in various applications of regenerative medicine, as well as analyzes the peculiarities of their use in different bioprinting methods to obtain high-quality tissue constructs.

## Multipotent mesenchymal stromal cells

The main cell type for using in regenerative medicine is multipotent stromal cells (MSCs), which were first described by Alexander Friedenstein as a population of bone marrow stem cells capable of differentiating osteogenically [4]. The “multipotent mesenchymal stromal cells” term was introduced after Arnold Kaplan’s team studies [5]. Many studies show that the MSC population is heterogeneous in its characteristics and varies greatly depending on the source tissue [6]. However, the role of MSCs in both natural regeneration and tissue engineering is indisputable. It is wellknown that MSCs are characterized by pronounced “homing”, that is tropism to damaged sites, and also have anti-inflammatory and immunosuppressive properties [7]. Moreover, MSCs slightly express major histocompatibility complexes, thus their transplantation does not lead to a host’s immune response and rejection, which makes them a convenient cell source for regenerative medicine [8].

According to generally accepted criteria, MSCs should have fibroblast-like morphology, adhere to plastics, express CD105, CD73, CD90 surface markers and differentiate osteogeniclly, chondrogenically, and adipogenically [9]. The main sources of MSCs are bone marrow, adipose tissue, amniotic fluid, umbilical cord blood, and Wharton’s jelly of the umbilical cord [10]. Depending on the tissue source, MSCs demonstrate different capabilities for differentiation and proliferation. It has been shown that bone marrow MSCs have the greatest potential for osteogenic differentiation [11], while adipose tissue MSCs have a predominantly angiogenic potential. MSCs obtained from umbilical cord blood have the highest proliferative potential, which allows them to maintain in culture for a long time [12, 13]. Along with classical sources of MSCs, dental pulp, gums, and periodontal ligament are accessible and promising sources of MSCs [14, 15]. MSCs from the mentioned sources have a higher potential for differentiation osteogenically and chondrogenically compared to MSCs from adipose tissue [16]. Thus, depending on the study objectives, researchers should choose a specific source of the MSC population.

## Epithelial cells

Epithelial tissue is a population of epithelial cells with apicobasal polarity which are closely bonded and connected with tight junctions and line the surfaces and cavities of most body organs. Epithelial tissue has a high regenerative potential and performs protective, secretory, transport, absorption, and other functions [17]. Epithelium is available in many human biological systems, such as the skin, cornea as well as reproductive, urinary, digestive and respiratory systems [18–20]. Primary cultures of epithelial cells are widely used to study cell differentiation and adhesion, absorption, permeability, and tissue regeneration mechanisms [21]. They are also used in the analysis of homeostasis metabolic disorders, such as the formation of fibrous tissue during healing and epithelialmesenchymal plasticity in case of malignant neoplasms [22, 23]. Moreover, epithelial cell cultures are widely used to study cytotoxicity of medications and chemicals.

In addition to primary cultures, there are multiple commercially available cultures of human epithelial cells obtained from various sources, such as bronchi, lungs, trachea, placenta, bladder, mammary glands, proximal tubules of kidneys, vascular endometrium, retinal pigment epithelium of the retina, etc. Depending on the study objectives, a particular source of epithelial cells is chosen.

Restoration of barrier epithelial tissues is an important and promising objective for tissue engineering. Bioequivalents used to facilitate tissue regeneration or *ex vivo* testing can be cell sheets formed from epithelial cells, as well as tissue-engineered constructs of various compositions created by 3D bioprinting methods [24, 25].

## Advantages of 3D culturing

The most promising approach to studying differentiation and intercellular interaction under conditions close to the same in native tissue may be the use of a spheroid in which a gradient of growth factors, nutrients, and oxygen is formed [26], and interaction between cells is enhanced by increasing the number of adhesive contacts [27] and bonds with newly synthesized ECM [28]. 3D cell cultures in the form of spheroids are close to native tissue in implementation of a multicellular microenvironment, signaling pathways, and intercellular interactions, which allows using them to model processes in native tissues [29].

The advantages of 3D culturing have been shown for many cell types. For instance, hepatocytes viability in spheroids is higher than in a monolayer; in 3D culture they retain a high capacity for detoxification [30]. In endothelial cell spheroids, vessel lumens are formed by apoptosis of central cells, which allows studying vasculogenesis [31]. Moreover, cell spheroids are widely used in oncological studies to analyze morphological changes in transformed cells [32].

The advantages of 3D culturing have been repeatedly noted in studies with MSCs involvement. In case of monolayer culturing, MSCs quickly age, their genetic instability accumulates, which limits the time of culturing, whereas the production of paracrine factors, differentiation potential, and the ability to restore recipient tissues after transplantation decrease [33]. Under three-dimensional conditions, the culture can be maintained for a long period (up to 7 months) [34]. This phenomenon can be explained, inter alia, by the increase in the expression of *Oct4A* and *Nanog*, *SOX2*, *SSEA-4*, *TRA-1-60*, and *TRA-181* pluripotency markers in spheroids. This explains the long-term preservation of cell stemness in spheroids compared to a monolayer [35]. It has been shown that after spheroid dissociation with a trypsin solution, the cells proliferate more actively than the cells of a monolayer culture. Thus, 3D culturing also increases the proliferative potential of cells [36].

Reactivation is the process of activating the spheroid cells proliferation and migration when the spheroid is placed in 2D conditions on an adhesive substrate [2]. It has been shown that secretory activity in spheroids increases and, hence, the MSCs anti-inflammatory potential grows due to such cytokines and growth factors as VEGF (vascular endothelial growth factor), basic fibroblast growth factor, angiogenin, procathepsin B, and interleukin-11 [37].

3D culturing also increases the differentiation potential and sensitivity of cells to inducers, which is important for regenerative medicine. For example, when exposed to VEGF, spheroids from MSCs differentiate angiogenically faster than in a monolayer culture [38, 39]. 3D culturing of human umbilical vein endothelial cells (HUVEC) stimulates the expression of such angiogenesis genes as *CD31*, *VEGFR-1*, *VEGFR-2*, as well as Tie-1 and Tie-2 angiopoietin receptors, an important growth factor that stimulates angiogenesis [40]. In spheroids from MSCs of adipose tissue, as well as of the periodontal ligament, the efficiency of osteogenic differentiation increases, which is confirmed by an increase in the synthesis of alkaline phosphatase, osteocalcin, and mineralization [41, 42]. Effective differentiation is explained by a high level of ECM in the spheroid, which promotes signaling through α2β1-integrin receptors, and which is required for osteogenesis [43]. In 3D culture, the myogenic potential of MSCs also increases compared to the same in a monolayer. Mature structures, myofibrils, are formed in spheroids, whereas in 2D cultures only the initial stages of spontaneous myogenic differentiation (expression of the MyoD marker) are observed [44]. It has also been shown that spheroids have a higher regenerative potential compared to a monolayer culture and retain their differentiation potential longer after transplantation into the damaged area [43]. Exemplified by cranial bone injury, it was demonstrated that when a bone marrow MSC suspension is transplanted into the damaged area, after 4 weeks, the damaged area is mainly filled with fibrous tissue whereas spheroid transplantation promotes formation of high-quality bone tissue [45]. Similar results were seen for MSCs isolated from the periodontal ligament and adipose tissue [42, 46].

The advantages of 3D culturing have also been shown for epithelial cells. It is known that primary cultures of epithelial cells, when cultivated under 2D conditions, lose their true epithelial phenotype, going through an epithelial-mesenchymal transition and acquiring a mixed phenotype. 3D cultures of spheroids allow to restore the cell phenotype due to the reverse mesenchymalepithelial transition [47–49]. When placed in 3D culture, mesenchymal cells also partially go through a mesenchymal-epithelial transition during epithelialization of the surface area of spheroids [50]. Moreover, the creation of 3D spheroid cultures from epithelial cells allows forming a more natural microenvironment due to cellular interactions and contacts with the ECM, increasing the viability of epithelial cells, migration, and secretory activity compared to 2D cultures [51, 52].

Organotypic models of malignant tumors of epithelial origin are often successfully obtained and used for future research and diagnostics in the form of cell spheroids [53, 54]. Spheroids from tumor cells allow discovering peculiarities of tumor invasion of various cancer types. It has been established that co-culturing of tumor cells of the epithelial phenotype with MSCs enhances the change in phenotype from epithelial to mesenchymal. Nevertheless, this allows getting a positive effect in forming a more accurate model of breast cancer invasion [53]. In case of colon cancer cells, researchers found the cells gaining invasive phenotype in three-dimensional cultures with additional stimulation by epithelial and crypt growth factors, although such a transition of the phenotype was not seen in a 2D culture [55].

## Spheroids formation

**Figure 1.**
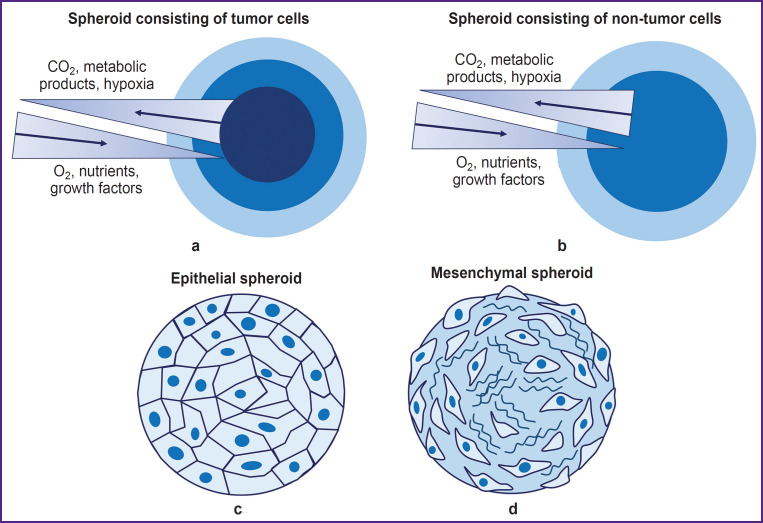
Structure of spheroids formed from tumor (a) and non-tumor (b) cells, from cells of epithelial (c) and mesenchymal phenotypes (d)

Spheroids can be formed from various cell cultures: cells of tumor and non-tumor nature, mesenchymal, and epithelial cell phenotypes (Figure 1). The morphology of spheroids as well as the efficiency of their formation depend on the cells type and morphological and functional characteristics. Non-tumor spheroids have 2 areas: external and internal. Spheroids from cells have 3 areas: proliferative external cortex, neutral silent area, and necrotic core [56]. Spheroids from cells of epithelial phenotype are characterized by densely packed cells with well-developed cytoskeleton and many intercellular contacts in the internal area, whereas spheroids from cells of mesenchymal phenotype are characterized by a densely packed surface area of epithelial-like cells and a loose internal area with a lot of ECM [57]. Typically, by day 7 of culturing, cells completely stop proliferating [39], except for spheroids formed from cancer cell lines [58, 59].

Spheroid formation is typically divided into the following stages: migration, aggregation and adhesion, compaction [60]. At first, individual spherical cells move randomly with the help of short processes — filopodia, then the cells begin to interact due to adhesive contacts, thus forming aggregates. This process takes approximately two hours [61]. Then compaction starts, and the main role there is given to E-cadherin, actin, and tubulin of microtubules [60]. Various compounds that selectively block polymerization of structures in cells are used to establish the impact of the main molecules of the cytoskeleton. Cytochalasin D (a blocker of fibrillar actin polymerization) is usually used to destroy the actin cytoskeleton [62], whereas antibodies to E-cadherin, integrins, and connexins are used to destroy cell adhesion proteins. Nocodazole and colchicine are used to block polymerization of tubulin microtubules. During spheroid formation, the cytoskeleton undergoes significant changes. In general, in 3D cultures, actin microfilaments of the cell are located in the cortical layer of the cytoplasm [63]. It has been shown that hardness decreases in the surface cells of spheroids, compared to a monolayer culture [64]. Cytochalasin D has different effects on spheroids from various cell types. For example, spheroids from bone marrow MSCs can be formed in presence of cytochalasin D, but they become looser, their diameter is 133% larger compared to the control [65], whereas treatment of epithelial spheroids with cytochalasin D, even in very low concentrations, completely terminates their formation [66]. It has been shown that treatment of chondrospheres with cytochalasin D seriously affects the dynamics of cell fusion and spreading and significantly reduces the spheroids mechanical properties. At that, destruction of microtubules with nocodazole significantly affects spheroids reactivation and unexpectedly results in an increase in hardness. Destruction of intermediate filaments (vimentin) also affects reactivation [67].

Damage of the cytoskeleton and intercellular contacts has the greatest impact on formation of epithelial spheroids, which is explained by the peculiarities of their structure and functioning of natural epithelial tissues due to a large number of intercellular contacts [68, 69]. The study of factors impacting intercellular adhesion of cells of the epithelial and mesenchymal phenotypes allows researchers to foresee the distribution in spheroids during self-organization [68, 69], as well as to intentionally influence sorting and separation during spheroidogenesis and formation of cultivated tissues [70, 71].

The contribution of different cytoskeleton and ECM components to the mechanical properties of spheroids primarily depends on the cell type. The analysis of the influence of cytoskeleton molecules and adhesion on formation of spheroids from cells of the epithelial and mesenchymal phenotypes is shown in Table 1 [65, 67–76]. The data indicate that, subject to various blockers usage, it is possible to influence the mechanical properties of spheroids and, thus, their behavior after bioprinting.

**T a b l e 1.** Role of cytoskeleton elements and adhesion molecules in formation of epithelial, mesenchymal, and mixed spheroids

Spheroid type	Impact and impact’s effect	Cell type	Impact on spheroidogenesis (+/±/-)	References
***Actin cytoskeleton***				
Epithelial	Cytochalasin D (inhibits polymerization of positive ends of fibrillar actin)	Rat hepatocyte culture	±; normal spheroid formation occurred at a concentration of 1 μM, but at a concentration of over 10 μM, formation was breached	[72]
		Primary culture of male rat hepatocytes	–; proper spheroids were not formed, only individual cell clusters formed	[73]
Mesenchymal	Cytochalasin D (inhibits polymerization of positive ends of fibrillar actin)	Bone marrow MSCs	±; spheroids were formed but were 133% larger than the controls	[65]
		Primary sheep chondrocyte culture	±; at a concentration of 10 μM spheroids formed but they were loose and fragile. The surface was covered with balled cells, spheroids lost the ability to fuse, and cells lost the ability to proliferate and migrate after moving spheroids in 2D conditions on an adhesive surface	[67]
***Adhesion molecules (cadherins, connexins, zona occludens molecules, integrins, etc.)***				
Epithelial	Antibodies to adhesion molecules (against E-cadherin and connexin 32)	Rat hepatocyte culture	–; spheroid formation was significantly impaired at the aggregation stage	[72]
	Antibodies to adhesion molecules (against ZO-1 molecules)		+; spheroid formation was normal	
	Knockdown of E-cadherin, α-catenin, and P-cadherin	Colorectal cancer (SW620, DLD-1, HCT116)	-; colorectal cancer cells that lost E-cadherin, α-catenin, and P-cadherin could not form spheroids and participate in aggregation with cells that have the said molecules	[69]
	Knockdown of E-cadherin	Breast cancer (MCF10A)	±; cells without E-cadherin encapsulated in a (collagen–alginate) matrix with increased hardness could not migrate from mixed spheroids	[68]
Mesenchymal	Impact on cadherin (E, N, P) expression by SynNotch signaling	Mouse fibroblasts (L929)	+; spheroids underwent cellular reorganization when activating various cadherins via the synNotch molecular cascade	[74]
***Microtubules***				
Epithelial	Nocodazole and taxol	Primary rat hepatocyte culture	+; spheroid formation was not impaired, despite the disordered organization of the tubulin cell networks	[73]
	Colchicine	Rat hepatocyte culture	±; normal spheroid formation occurred at a concentration of 0.5 μM, but at a concentration of over 5 μM, irregular aggregates were formed	[72]
Mesenchymal	Nocodazole	Primary sheep chondrocyte culture	±; spheroids were formed normally, one surface was filled with partially balled cells, cell migration was impaired after placing the spheroids in 2D conditions on an adhesive surface (1 μM)	[67]
***Other molecules and factors impacting cell adhesion***				
Epithelial	Okadaic acid (inhibits specific protein phosphatases)	Rat hepatocyte culture	±; spheroids were not formed at a concentration of 3–30 nM, but at a lower concentration (0.3 nM), the formation was not impaired	[72]
	Lectins		±; depending on the type of lectins, the formation of spheroids was different, formation was impaired for some cultures	
	Inhibitors of glycosphingolipids and proteoglycans synthesis		+; no impairment of spheroid formation was revealed	
	Hardness of environment	Breast cancer (MCF10A, MCF7)	±; mixed spheroids (MCF10A and MCF7) in a matrix (collagen–alginate) of increased hardness underwent cell sorting and separation	[68]
Mesenchymal	WFA (inhibitor of intermediate filaments)	Primary sheep chondrocyte culture	±; the spheroids formation was successful, but cell migration from the spheroid to the monolayer was impaired (1 μM)	[67]
Epithelial/ mesenchymal	DNA hybridization	Breast cancer epithelium (MDA-MB-468), fibroblasts (NIH/3T3)	+; spheroids from cells conjugated with complementary DNA molecules were more successful in forming a mixed culture, as well as in synthesizing of significantly more fibronectin	[70]
	Y-27632 (the ROCK inhibitor)	Langerhans pancreatic islet cells and MSCs	+; when adding the ROCK inhibitor (30 μm), mixed spheroids were successfully formed, in the control group, sorting and separation of cells were observed	[75]
	Surface tension	Neuronal cells and glia	+; surface tension and the interacting force between cells contribute to cells sorting of in spheroids	[71, 76]

N o t e s: “+” in the “Impact on spheroidogenesis (+/±/–)” column means the formation of spheroids despite any impact; “±” — formation occurred with the described violations; “–” — spheroids were not formed.

## Mechanical properties of spheroids

During growth, the spheroid actively synthesizes various ECM components, thus forming mechanical stress. The cell can determine the hardness of the microenvironment by binding to the ECM through various contacts and components of cytoskeleton [77]. The bond is ensured mainly by focal contacts, which consist of integrin receptors on the membrane surface and connect the actin cytoskeleton to the ECM [78]. Mechanical changes can lead to activation of integrinassociated kinases and trigger various signaling cascades, such as ERK, JNK, Wnt-catenin, and Hippo [79]. The Hippo signaling pathway is involved in mechanotransduction, that is it translates mechanical signals to the gene expression level [80]. When cells are cultured in a soft matrix, the YAP/TAZ component of the Hippo cascade is located in the cytoplasm, and in case of increased hardness, the complex is translocated into the nucleus, changing gene expression and affecting cell differentiation, and proliferation [81]. Thus, matrix hardness directly affects cellular differentiation. For example, it has been shown that during MSCs culturing on a softer substrate, the cells differentiate adipogenically, whereas a harder substrate stimulates the development of osteocytes [82]. A hard substrate affects osteogenic differentiation through mechanosensitive genes. Cell compression and stretching blocks myogenic and adipogenic differentiation by suppressing the expression of the *MyoD* and *PPAR-γ* genes, and simultaneously activates the expression of the *Runx2*, *Osterix*, *Msx2*, and *Sox9* genes, stimulating osteogenic differentiation of MSCs [83].

Many ECM proteins accumulate growth factors. For instance, fibronectin binds VEGF [84]. Some ECM components can activate receptors. For example, laminins, fibrillins, and thrombospondins contain a domain similar to the epidermal growth factor, which can bind to the corresponding receptor [85]. Thus, due to the accumulation of ECM, a favorable mechanical environment for targeted differentiation is formed in spheroids.

Currently, methods for measuring the mechanical properties of microobjects are being actively developed; the key role of biomechanical interactions in the organism development as well as in pathological and regenerative processes has been established. The impact of substrate hardness on the growth and differentiation of stem cells has been shown [86], lower hardness of cancer cells compared to normal cells has been proved [87], and the important role of mechanical aspects in embryonic development is being studied.

Young’s modulus, measured in pascals (Pa), is generally used to characterize the hardness of a material. Nanoindentation and atomic force microscopy are commonly applied to determine local properties of tissue, as well as monolayer culture or surface cells of spheroids [88]. The method is limited to measuring surface properties with a penetration depth (indentation) of up to 1 μm. For macrosamples (with a thickness ranging from a few millimeters to several centimeters), mechanical tests are used for stretching, compression, and bending of the material, which allow obtaining average mechanical properties for the sample [89, 90]. Systems that allow cell aggregates and cell layers to be analyzed using this method have been developed.

Moreover, the parallel plate compression method on the MicroSquisher device [91] and cavitation rheology [92] are used to determine the hardness of a cell sheet, spheroid, or tissue in general. These methods allowed to establish that a monolayer culture of MSCs cultivated on plastic has, on average, a Young’s modulus measured in gigapascals (GPa), whereas in spheroids the hardness of surface cells does not exceed 0.1 kPa. Atomic force microscopy revealed that the Young’s modulus of spheroids from the mouse fibroblast 3T3 cell line was within the range of 0.3–3.5 kPa [93], and from the epithelial cell line of human colon adenocarcinoma (LS174T) — within the range of 0.3–0.6 kPa.

The cavitation rheology method was used to analyze the mechanical properties of spheroids from the transformed HEK 293 cell line. The critical pressure required to break the bond between cells in such spheroids was 0.013–0.500 kPa [94]. The MicroSquisher device helped to determine that the Young’s modulus of three-day spheroids from MSCs was 42.28±6.14 Pa, and the same modulus of seven-day spheroids was 62.40±5.58 Pa [95]. The analysis of mechanical properties for soft biological tissues or bioequivalent components is conducted using a rheometer, which measures such parameters of gels and liquids as the shear modulus and viscosity [96].

To analyze the mechanical properties and surface tension of spheroids, a researcher can use the aspiration method, which involves absorption of a single spheroid with a micropipette followed by calculation of the required parameters according to the Young–Laplace equation, which describes the relationship between the internal pressure of spheroids and the cellular environment through a curved interface [97]. This method was used for comparison of the mechanical and viscoelastic properties of spheroids of different sizes obtained from HUVEC, 3T3, mouse breast cancer cells (4T1), human skin fibroblasts (HDF), and a co-culture of human MSCs and HUVEC [98]. Spheroids from fibroblasts and MSCs compacted faster and formed more ECM on the surface compared to epithelial spheroids. This method was also applied to demonstrate that the surface tension of spheroids from HUVEC, 3T3, 4T1, HDF, MSC/ HUVEC, and MSC after 2 days of culturing was ~14, 30, 37, 41, 51, and 66 mN/m, respectively. In other words, the surface tension of MSCs-only spheroids is approximately five times higher than the same value of spheroids from endothelial cells. It was also established that the surface tension of 4T1, HDF, and MSC/HUVEC spheroids increases over time, whereas the tension of spheroids obtained from other cell types remains virtually unchanged. A positive correlation between the surface tension of spheroids and their compactness was revealed [99]; it depends on the amount of collagen [100]. The hardness of a spheroid largely depends on the composition and structure of the ECM, which can have an elasticity modulus in the range from several megapascals (elastin) to several gigapascals (collagen) [101]. At that, the mechanical properties can also vary significantly depending on the orientation, cross-linking, and interaction of different types of fibers [102].

Since spheroids are widely used to model tissue morphogenesis, regeneration, and metastasis, and simultaneously they serve as building blocks in bioprinting, the study of their mechanical properties is an important task for tissue engineering. The surface tension and hardness of spheroids have a great impact on their capacity to fuse, as well as on reactivation, that is the capacity of cells to migrate from the spheroid after its placement on an adhesive substrate or in hydrogel [103, 104]. These parameters, in turn, are directly associated with the maturation rate of the printed construct [105–108]. Changing the mechanical properties of spheroids by modeling cytoskeletal components with specific blockers can increase the efficiency of fusion and reactivation of spheroids after bioprinting [109].

The mechanism of fusion of cell aggregates is usually considered similarly to the mechanism of fusion of liquid droplets [110]. According to this model, the main parameters determining the mechanism and rate of fusion are viscosity and surface tension [111], which in cell spheroids is determined by cell adhesion molecules, the cytoskeleton, and the ECM.

There are studies aimed at determination of the relationship between the mechanical properties of spheroids and the rate of their fusion [94, 112, 113], but the results thereof are somewhat contradictory. For example, it was established that the tension of the surface cells in spheroids from fibroblasts promotes their faster fusion compared to spheroids from epithelial cells (Chinese hamster ovary CHO cell line) [112]. Other researchers claim that the fusion of spheroids from epithelial cells (retinal pigment epithelium) is faster than the fusion from mesenchymal cells isolated from the eye limbus [113]. Probably, the high ECM content and dense cell packing in the outer layer of MSC spheroids negatively affect their migration and, thus, the fusion of spheroids. At that, three-day spheroids from MSCs fuse faster, which is associated with a low ECM content. Similar results were obtained for spheroids from sheep chondrocytes. At early stages of culturing, they fuse faster than at later stages due to ECM accumulation [114]. More efficient fusion of spheroids from epithelial cells may also be due to the fact that collective migration is more typical of epithelial cells with well-developed intercellular contacts [115].

**Figure 2.**
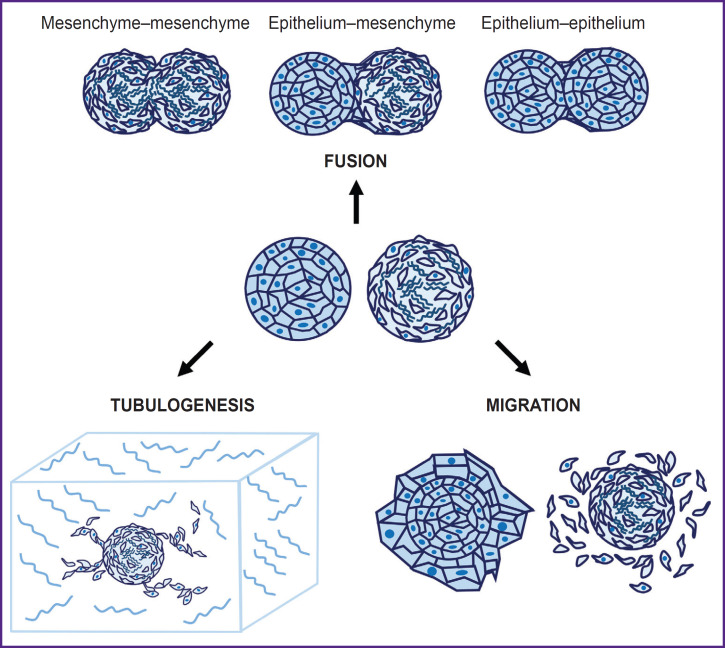
Patterns of bioequivalent maturation after bioprinting

In the context of tissue engineering, one shall often combine several cell types or spheroids in a construct so that it best reproduced the tissue structure *in vitro*. This is achieved by using spheroids of different cell types, and cells can be added sequentially to form organized layers. For example, adding a suspension of epithelial cells to pre-formed mesenchymal spheroids allows creating an *in vitro* model for studying embryogenesis and epithelialmesenchymal plasticity [3, 116], which form the basis of many morphogenetic processes, as well as tissue regeneration and metastasis [117]. It is also possible to separately use spheroids of mesenchymal and epithelial cells for bioprinting; their layer-by-layer separation occurs as the construct matures. It has been established that fusion of mesenchymal and epithelial spheroids leads to epithelial cells overgrowing mesenchymal cells [113]. The study of mechanical properties of spheroids of different phenotypes requires further research, since they directly affect the way of bioequivalent maturation after bioprinting [47], namely the rate of spheroids fusion, cell migration, and processes formation. The main ways of bioequivalents maturation after bioprinting are shown in Figure 2.

## Bioprinting with spheroids

**Figure 3.**
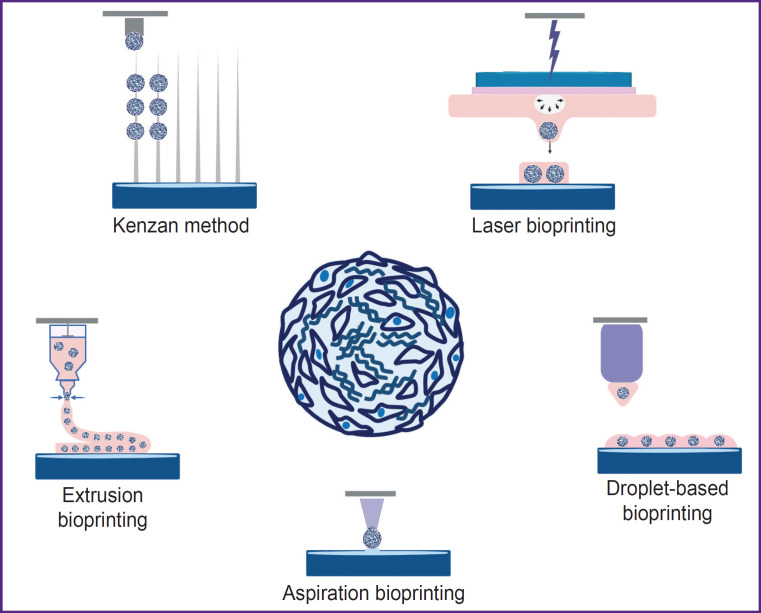
Methods of bioprinting using spheroids

Three-dimensional bioprinting has many advantages and allows creating complex tissues consisting of several layers of different cell types in the corresponding ECM [118, 119]. By present, many different bioprinting methods have been developed, for instance, extrusion, aspiration, laser, droplet-based, etc. [120, 121] (Figure 3). The method choice depends on the desired morphology and size of the bioequivalent, the hydrogel used, as well as the phenotype of the cells and their mechanical properties. The use of spheroids as building blocks takes the attention of an increasing number of researchers [122, 123], as spheroids have increased regenerative capacity compared to monolayer culture, better imitate the physiological conditions typical of the native tissue, and are characterized by high viability.

Complete filling of the printed construct can be achieved both by cell migration from the spheroid and by their fusion. These parameters directly depend on the mechanical properties of the spheroids [57], the hydrogel used, and the distance between them [124]. For example, it was shown that when spheroids from adipose tissue-derived MSCs are located maximum 100 μm from each other, they are more likely to fuse, whereas when the distance is over 400 μm, they form processes reaching towards each other [124]. HUVEC spheroids located at a distance of 800 μm form a more branched network of processes (total length of the processes is 11 mm) compared to the group where the distance between spheroids is 3000 μm (6 mm) [105]. The degree of differentiation can also have a significant impact on the rate of fusion. For example, osteogenically differentiated spheroids from MSCs fuse more slowly compared to undifferentiated ones [106]. On day 5 of culturing these spheroids fuse significantly better compared to day 7 spheroids, which is also important to consider when creating cartilage equivalents [107]. Fusion of spheroids from cardiomyocytes and fibroblasts occurs on day 4 of culturing with a distance of approximately 50 μm between them [105].

There are some difficulties associated with bioprinting with spheroids, primarily related to the aggregation of spheroids and clogging of the nozzle. This issue can be solved by choosing a special hydrogel composition and bioprinting method, as well as by reducing the size of spheroids and modifying their mechanical properties. A comparison of bioprinting methods and the characteristics of the spheroids used is given in Table 2 [98, 105–108, 125–137].

**T a b l e 2.** Bioprinting methods and their use for bioequivalents creation

Model	Cell type	Hydrogel type	Model characteristics	Results	References
***Extrusion bioprinting***					
Breast cancer	Breast cancer cells MDA-MB-231 Adipose tissue MSCs	Hyaluronic acid	Bioprinting of adipose tissue bioequivalents with 228±22 μm MSC spheroids followed by adipogenic differentiation followed by application to the surface of the breast cancer cell construct	A bioequivalent was created to study the interaction of cancer cells and adipose tissue in a human breast cancer model. The ability of cancer cells to decrease the lipid content and remodel ECM in adipose tissue was demonstrated	[125]
	Breast cancer cells MCF10A, MCF10A-NeuN, MDA-MB-231, MCF7 HUVEC	Matrigel Gelatin–alginate Collagen– alginate	Layer-by-layer bioprinting with spheroids of ~70 μm at a concentration of 120– 150 spheroids/cm^2^ from mammary epithelial cells and endothelial cells	A bioequivalent was created to study the interaction between mammary epithelial cells and vascular endothelial cells. It was shown that co-culturg in the printed construct is more resistant to paclitaxel compared to the monoculture of cells	[126]
Cartilage tissue	MSCs	Xanthan gum + alginate	Bioprinting with spheroids from MSCs of ~180 μm, consisting of ~3048 cells, at a concentration of 2500 spheroids per 1 ml of hydrogel followed by their chondrogenic differentiation for 28 days	A bioequivalent of cartilage tissue was created, its chondrogenic differentiation and viability for 56 days of cultivation were shown, an optimal method for mixing bioink to reduce the risk of spheroid destruction and their uniform distribution in the hydrogel was described. It was demonstrated that the described spheroids completely fused after 46 h; it was established that it is better for bioprinting to use spheroids on the day 5 of cultivation rather than on day 7	[107]
Liver	Primary mouse hepatocytes	Decellularized matrix obtained from pig liver + gelatin + hyaluronic acid + CaCl_2_ Hyaluronic acid + alginate + gelatin	In the first stage, spheroids of 160-220 μm from hepatocytes were obtained and mixed with decellularized liver matrix. Then, the spheroids were printed with a 120 μm nozzle using a hydrogel based on hyaluronic acid, alginate, and gelatin	A functional *in vitro* liver model was obtained to assess the toxicity of medications. It was demonstrated that the inclusion of spheroids in the liver matrix facilitates obtaining a more adequate model, as it increases the sensitivity of the bioequivalent to hepatotoxic medications, as well as stimulates the expression of liver genes	[127]
Cartilage tissue	Rabbit auricle chondrocytes	Gelatin methacrylate (GelMA) 10% Polyethylene oxide 1%	Spheroids from chondrocytes containing 500 cells in cylindrical constructs (diameter 10 mm, length 2.5 mm), 275 spheroids per construct	A functional equivalent of cartilage tissue was obtained; 4 weeks after transplantation, it stimulated deposition of glycosaminoglycans and type II collagen. After 12 weeks, the implanted equivalent had a structure similar to natural cartilage tissue with typical chondrocyte morphology and extracellular matrix	[128]
Liver	Endothelial cells C166 Primary mouse hepatocytes	Gelatin methacrylate (GelMA)	Bioprinting of self-organized spheroids from hepatocytes and endothelial cells on a preformed polymer framework having analogs of vessels populated with spheroids of endothelial cells	A functional vascularized liver bioequivalent was obtained; it expressed typical markers, as well as maintained viability and stimulated neovascularization for two weeks after its transplantation into mice	[129]
Thyroid gland	Thyrocytes Endothelial cells (of allantois)	Collagen	Spheroids from thyrocytes and endothelial cells of approximately 390 and 490 μm, respectively	A functional vascularized thyroid equivalent was obtained; it was capable of normalizing the level of blood thyroxine and body temperature after implantation under the renal capsule in hypothyroid mice	[130]
***Laser bioprinting***					
Cartilage tissue	Human periosteum cells	Collagen	Bioprinting with 150 and 300 μm spheroids from human periosteum cells, which were pre-differentiated chondrogenically for 3, 7, and 14 days	A multilayer bioequivalent of cartilage tissue was obtained. It was demonstrated that 150 μm spheroids, pre-differentiated chondrogenically for 7 days, are optimal for laser printing. Optimal laser settings were chosen to reduce spheroid degradation. A target and shoot system was developed; it allows using spheroids of about 300 μm for bioprinting, as well as conducting targeted choice of spheroids from a suspension for printing	[131]
Spheroids in hydrogel	Human cord blood-derived MSCs	PEG-fibrin + hyaluronic acid	Bioprinting with 150 μm spheroids	An analysis of the settings impact and methods of laser transfer of spheroids on the spheroids’ viability was performed	[132]
***Aspiiration bioprinting***					
Bone tissue	Bone marrow MSCs THP-1 monocytes differentiated into osteoclasts	Alginate	Bioprinting with spheroids from MSCs (400-800 μm) differentiated for 28 days osteogenically, as well as spheroids from a combined culture of MSCs and monocytes (2:1 and 3:2) (250-600 μm), simultaneously differentiated in the direction of osteoblasts and osteoclasts. A 200 μm nozzle was used for aspiration	A bioequivalent of bone tissue was obtained to study bone remodeling. The optimal composition of the microgel based on alginate was specified to reduce the deformation of spheroids caused by the mechanical characteristics of the hydrogel; it was also used to increase the rate of spheroids fusion. It was demonstrated that osteogenically differentiated spheroids fuse slower	[106]
Cartilage tissue	MSCs		*Strategy 1.* Bioprinting with 3 day spheroids from MSCs (150-450 μm) followed by construct extraction on day 4 and chondrogenic differentiation for 20 days *Strategy 2.* Bioprinting with 3 day spheroids from MSCs, pre-differentiated chondrogenically for 19 days, followed by bioprinting and construct extraction from the hydrogel	A bioequivalent of cartilage tissue was obtained. It was demonstrated that the most effective approach involves preliminary differentiation of MSC spheroids chondrogenically before their use for bioprinting. This is due to the fact that the ECM accumulation during such differentiation results in an increase in spheroids, as well as the surface tension of the cells, which is critically important in aspiration bioprinting, as it affects the safety of spheroids. It was also shown that here a more mature cartilage tissue is obtained compared to the equivalent obtained when printing with MSC-spheroids	[108]
Bone tissue	MSCs		*Strategy 1*. Spheroids from MSCs differentiated osteogenically for 14 days *Strategy 2.* Differentiation of MSCs in a monolayer (7 days) followed by differentiation in a spheroid (7 days) *Strategy 3.* Differentiation of MSCs in a monolayer (12 days) followed by differentiation in a spheroid (2 days)	A bioequivalent of bone tissue was obtained. It has been shown that the use of osteogenic differentiated spheroids for bioprinting is an ineffective approach, since the accumulation of ECM negatively affects their fusion ability. The optimal strategy is osteogenic differentiation of MSCs in a monolayer and then short-term 3D cultivation before bioprinting. In this case, spheroids fuse better and form a holistic and more mature bone bioequivalent	[108]
Heart	iPSCs Cardiac fibroblasts Bone marrow MSCs	Modified hyaluronic acid	MSC spheroids 200–400 μm in size (5000 and 10,000 cells, respectively) Cardiospheroids (5000 cells) obtained by mixing cardiomyocytes from iPSCs and fibroblasts at a ratio of 4:1 to model healthy and 1:4 to model fibrous cardiac tissue	A functional model of focal cardiac fibrosis was developed. Due to precise bioprinting, cardiac microtissue was obtained by fusing healthy and fibrous spheroids in a specific ratio. The impact of fibrous scars on the electrophysiological properties of the tissue was demonstrated. Microtissue from healthy spheroids had an increased contraction amplitude compared to fibrous tissue. The possibility of using this model for testing microRNA-based medications was demonstrated. Hydrogels that were locally destroyed during printing were used, which reduced the shear stress exerted on the spheroid and, thus, reduced their deformation. After printing, such hydrogels can restore their structure. When printing spheroids at a distance of ~50 μm, the spheroids fused after 4 days. The hydrogel properties allow the mature construct to be extracted from it without any mechanical damage	[105]
Bone tissue	MSCs HUVEC	Fibrin	Spheroids from MSCs and HUVEC in a 1:1 ratio, approximately 400 μm in size *Strategy 1.* Bioprinting with 2-day-old spheroids, fusion for 3 days, followed by induction of osteogenic differentiation for 12 days *Strategy 2.* Spheroids were cultured in a standard medium for 5 days, then induced osteogenically for 10 days and used for bioprinting of constructs, which were also kept in an osteogenic medium for 2 days	A bone tissue equivalent was obtained. It was shown that the approach in which pre-differentiated spheroids reused for bioprinting is more effective, as it allows forming a more mature and stable construct	[98]
***Droplet-based bioprinting***					
Heart	Human cardiac fibroblasts Human cardiomyocytes (AC16)	Alginate	Cell suspension was used	A model was developed to assess electrostatic interactions between cardiomyocytes and fibroblasts in cardiac tissue	[133]
Liver	Hepatocytes from iPSCs Embryonic stem cells	Alginate	Cell suspension was used	Functional liver equivalents were obtained; they remain viable for 17 days of culturing and express liver markers such as albumin and HNF4a	[134]
Alveoli	Endothelial cells (EA.hy926) Alveolar epithelial cells type II (A549)	Matrigel	Cell suspension was used	An *in vitro* model of the lung alveoli consisting of endothelial cells, basement membrane, and epithelial cells was obtained	[135]
***Kenzan method***					
Vessels	HUVEC Aortic smooth muscle cells Human dermal fibroblasts	No hydrogel	Approximately 600 μm spheroids of human endothelial cells, aortic smooth muscle cells, and human dermal fibroblasts were threaded onto 170 μm diameter needles located at a distance of 400 μm from each other	A mechanically hard and mature vessel bioequivalent of 1.5 mm in diameter and 7 mm in length was obtained; it maintained its integrity after implantation in rats	[136]
Endo-metriosis	Endometrial epithelial cells (12Z) Immortalized stromal cells isolated from the uterus of a patient with non-malignant fibroids (T-HESCs) Ovarian cancer cells (HEYA8)	No hydrogel	Heterospheroids of 500 μm with epithelial cells (12Z) were seen on the surface and stromal cells (T-HESCs) in the central area of the spheroid Monospheroids from 12Z and T-HESCs	A functional model of endometriosis and endometriotic microenvironment *in vitro* was obtained	[137]

### Extrusion bioprinting

Extrusion is an earliest and simplest bioprinting method, which involves squeezing a viscous material from a bioprinter cartridge according to a pre-defined program that specifies pressure, speed, temperature, and a 3D bioprinting model. This is a fast and easily scalable method that allows getting constructs with high cellular density and maintain cell viability at a level of approximately 70–80% [138]. However, the resolution of such printing is about 100–200 μm, which is significantly lower than the same value of aspiration and laser bioprinting [139]. Compared to other technologies, this method is the most generalized and can be used with different types of spheroids and hydrogels. But it is not applicable to large spheroids, which can clog the nozzle, and mechanical compression leads to the surface cell death. Small spheroids are often unevenly distributed in hydrogel, and this results in their being located far from each other, thus they fuse worse and do not form the required construct structure.

The possibility of extrusion bioprinting with spheroids was first demonstrated in 2004 by a research group headed by Vladimir Mironov, a Russian scientist [140]. Since that time, this field achieved great success. For example, using spheroids from thyroid cells and allantois endothelial cells, a functioning mouse thyroid gland was created, which — after transplantation under the renal capsule — maintained the level of blood thyroxine and body temperature [130]. Particular attention is given to bioprinting of liver equivalents, for example, for testing hepatotoxic medications. A viable liver construct was obtained using spheroids consisting of endothelial cells, MSCs, and hepatocytes; the cells of the construct not only express liver proteins, but also produce urea and albumin, that is they maintain their functionality [141]. The study [127] demonstrated that mouse hepatocytes were pre-encapsulated in decellularized liver matrix and this resulted in formation of a mature and functional bioequivalent that was much more sensitive to hepatotoxic medications compared to conventional hepatospheroids.

Many studies are devoted to bioprinting of cardiac tissue. Scientists have already managed to achieve contractility of spheroids obtained from a combined culture of cardiomyocytes, endothelial cells, and fibroblasts [142]. A functional model of focal cardiac fibrosis was developed to study the tissue electrophysiological properties and test medications [105]. Spheroids from adipose tissue MSCs being part of printed constructs are used for soft tissue regeneration due to their support of angiogenesis [130, 143], as well as for bone tissue engineering [144, 145]. Spheroids from MSCs and HUVEC encapsulated in collagen and fibrin hydrogel form constructs that effectively differentiate osteogenically and form a branched vascular network compared to the suspension of the same cells in a hydrogel [146]. Bioequivalents of cartilage tissue were also obtained [107, 128]. Quite a lot of studies are addressed to models of various cancer diseases [147]. Researchers obtained a bioequivalent to study the interaction between cancer cells and adipose tissue [125], as well as between epithelial and endothelial cells [126] in a model of human breast cancer.

### Aspiration bioprinting

The principle of aspiration bioprinting is to capture a spheroid by aspiration and transfer it to an exact position in a framework or hydrogel. The spheroid placement error is maximum of 11–15% relative to its size [98]. This method allows working with spheroids of various sizes, as well as having different mechanical properties, as it allows choosing a specific aspiration force to avoid spheroid deformation. In 2020, there was a study conducted that described in detail the mechanism for choosing the aspiration force in bioprinting depending on the mechanical properties of spheroids (viscoelastic properties and surface tension). For this purpose, the mechanical and viscoelastic properties of 200–600 μm spheroids from human umbilical vein endothelial cells (HUVEC), mouse fibroblasts (3T3), mouse breast cancer cells (4T1), human skin fibroblasts (HDF), and a co-culture of human and HUVEC MSCs were analyzed, and the correspondence of these characteristics to the optimal aspiration force and bioprinting time was described [98].

The aspiration bioprinting method was successfully applied to create bone [106, 108], cartilage [108], cardiac [105], and other types of tissue. It was demonstrated that the most effective approach to obtain cartilage equivalents was the approach with the MSC spheroids chondrogenic pre-differentiation and further usage in bioprinting. This is due to the fact that the ECM accumulation during chondrodifferentiation results in an increase in the spheroid size as well as in an increase in the cells surface tension, which is critical in aspiration bioprinting, since it affects the safety of spheroids [108]. While getting a bone equivalent, it was found that the more effective model is bioprinting with undifferentiated spheroids from MSCs followed by their fusion and osteodifferentiation of the construct. This is due to the fact that a high ECM content significantly increases the surface tension in spheroid cells and does not allow them to fuse into a proper construct [108]. However, a more mature construct was obtained from osteogenically pre-differentiated mixed spheroids in the study [98], where a combined culture of MSCs and HUVEC was used to obtain a bone tissue equivalent.

Aspiration bioprinting was used in a recent study [105] to get a functional model of focal cardiac fibrosis by fusing healthy (cardiomyocytes) and fibrous (cardiomyocytes and fibroblasts) spheroids in a specific ratio. The impact of fibrous scars on the electrophysiological properties of the tissue was demonstrated, as well as the possibility of using this model for testing microRNA-based medications. Due to high accuracy of spheroid positioning during aspiration bioprinting, the researchers examined the impact of the distance between spheroids on their functional potential. It was found that HUVEC spheroids located closer to each other (400 μm) form a more branched network of processes compared to the group where the distance between spheroids is 3000 μm.

### Laser bioprinting

Laser bioprinting is mainly used for printing with cell suspensions [148] and provides high-precision manipulation of objects (up to 1 μm), usage of a wide range of bioink viscosities [149], and high cell viability after bioprinting. The technology based on laser-induced forward transfer (LIFT) was first successfully used for spheroid printing in 2023, when it was established that spheroids from human umbilical cord MSCs of approximately 150 μm can be printed with high precision using a hydrogel [132]. This study also analyzed the impact of laser transfer settings and methods on spheroid viability [132].

The principle of laser bioprinting is to create droplets with cells or spheroids that are transferred from the “donor” to the “recipient” by means of a laser pulse. The donor substrate is usually a glass slide coated with a thin metal layer that absorbs energy, as well as a layer of bioink (cells or spheroids with hydrogel). The laser energy is absorbed by the metal layer at the focus point. Here, the energy-absorbing layer heats up and evaporates, forming a bubble that pushes a stream of hydrogel with cells or spheroids out of the layer. Then, the droplet falls on the acceptor substrate, moving along the stream of bioink, and forms a construct. This method ensures high speed of printing, accuracy of cell movement, and cell survival. At that, the transfer conditions must be chosen depending on the type of hydrogel and cells [132].

This is a fairly new method, and thus there are few studies describing laser bioprinting with spheroids. There is another study dated 2024 where laser bioprinting was successfully used to create tissue equivalents of cartilage tissue with chondrogenically 7 days-predifferentiated spheroids from periosteal cells [131].

### Droplet-based bioprinting

Droplet-based bioprinting was first developed in the 2000s, when a research team led by Nakamura from the University of Toyama [150] optimized a traditional paper printer and made it compatible with cells and viscous hydrogel inks. The first constructs printed in this way were tubular structures consisting of HeLa cells. Dropletbased bioprinting has a high resolution comparable to laser bioprinting and is one of the simplest, fastest, and cheapest methods [151]. Moreover, droplet-based bioprinting can be used to apply bioink directly to the defect area by spraying droplets with cells, which is important for treatment of deep wounds, burns, and other surface defects [152].

At that, droplet-based bioprinting has many disadvantages. One of the most common issues is nozzle clogging. The nozzle diameter is usually 10– 150 μm, and thus this method is incompatible with the use of such large objects as spheroids >150 μm, as well as viscous hydrogels [153]. Therefore, in droplet-based bioprinting, cells are usually transferred as part of a standard nutrient medium and printed onto the surface of hydrogel [154]. Droplet-based bioprinting is also often used to get spheroids [155] and is similar in principle to the hanging drop method. Using droplet-based bioprinting, one can obtain spheroids of a specified size, immediately encapsulated in a certain hydrogel [156].

This method is very rarely used for bioprinting with spheroids, as they have a diameter exceeding the nozzle size, and the printing technology does not provide a construct with high cellular density and, thus, with high mechanical strength [157].

Despite the above-mentioned limitations, there are several cases of successful droplet-based bioprinting. For example, using an alginate-based hydrogel, functional liver bioequivalents were developed; they consisted of hepatocytes obtained from induced pluripotent stem cells (iPSCs) and human embryonic stem cells. 17 days after bioprinting, a functional construct expressing such liver markers as albumin and HNF4a was obtained [134]. Alginate hydrogel is optimal for droplet-based bioprinting, as it is a result of mixing two liquid components: alginate and calcium chloride, which form a viscous hydrogel when mixed. This solves the problem of nozzle clogging.

This study [135] used droplet-based bioprinting to obtain an *in vitro* model of lung alveoli consisting of endothelial cells, basement membrane, and epithelial cells. For this purpose, researchers printed a bioequivalent consisting of alveolar epithelial cells type II (A549) and endothelial cells (EA.hy926) separated by matrigel. It was demonstrated that, unlike the manual mixing method, bioprinting allows creating homogeneous cell layers.

Using the droplet-based bioprinting, the researchers obtained a model to assess electrostatic interactions between cardiomyocytes and fibroblasts in cardiac tissue, using two types of cells: cardiac fibroblasts and human cardiomyocytes [133].

However, in all the mentioned studies, a cell suspension was used for bioprinting, not spheroids.

### Kenzan method

Kenzan is a Japanese word that is literally translated as the “mountain from swords”. It is a holder made of many needles, designed to fix plants when creating flower arrangements. Japanese researchers suggested using similar structures to fix spheroids and get microtissues [158]. This method allows positioning spheroids on the surface of a pre-designed construct with an accuracy of up to 1 μm. The construct is a temporary support made of stainless-steel microneedles that can be removed after spheroids fusion and formation of a proper structure.

Currently, there is one commercially available bioprinter, the principle of which is based on the Kenzan method. This is Bio-3D Printer (Japan), which has microneedles of approximately 160 μm in diameter, which are located at a distance of 500 μm. The spheroids in such a system can contact and fuse only if their size is at least 400–600 μm. This method is very rarely used for bioprinting, although it is particularly suitable for producing tubular structures such as vessels, trachea, and urethra. In order to produce vascular structures with the Kenzan method, spheroids of approximately 600 μm in size from human endothelial cells, aortic smooth muscle cells, and dermal fibroblasts were threaded onto 170 μm diameter needles located 400 μm from each other. After four days of culturing, the spheroids fused to form a mature construct, and the needles were removed. The formed vessels were 1.5 mm in diameter and 7 mm in length. They were mechanically strong and maintained their structural integrity after implantation in rats [136].

There are several successful studies where this technology was used to create urethras and tracheas, but their results have not been published yet and have only been reported at conferences. Researchers in this study [137] obtained a functional model of endometriosis by using this method. At that, they used spheroids of about 500 μm in size from epithelial cells obtained from the uterus with endometriosis (12Z cell line), as well as from the ovarian cancer epithelial cell line (HEYA8).

## Conclusion

Bioprinting using spheroids as a cellular component of bioink is a promising area of regenerative medicine. In spheroids, cells form complexes of intercellular contacts synthesize extracellular matrix, intendedly differentiate more effectively and adapt to hypoxia [27–29]. Thus, bioequivalents formed with spheroids demonstrate much higher viability and functional differentiation potential compared to constructs that were printed using cell suspension [122, 140].

Maturation of bioequivalents after printing happens either due to spheroid fusion or by cell migration and processes formation. Either maturation type depends on the distance between the spheroids, as well as on their mechanical properties. Spheroids from adipose tissue MSCs fuse if they are located less than 100 μm from each other, whereas at a distance of over 400 μm they form processes towards each other [124]. Spheroids from endothelial cells also form a more branched network of processes at high printing density [105].

Spheroids from mesenchymal cells tend to accumulate extracellular matrix, so the longer their culturing period is, the worse they will fuse in the resulting bioequivalent [107]. Osteogenic and chondrogenic differentiation of MSCs leads to increased hardness and accumulation of extracellular matrix, thus such spheroids do not fuse after bioprinting. Many researchers recommend differentiating the tissueengineered construct after the primary maturation stage, that is after spheroids fusion. Here, a more integral structure with the microtissue properties is formed [106, 108]. However, in case of aspiration printing, on the contrary, it is better to use osteogenically and chondrogenically pre-differentiated spheroids, as the high surface tension allows protecting them from mechanical destruction during aspiration [108]. The same was established for laser bioprinting [131].

The most popular method is still extrusion bioprinting. It is a fast and easily scalable method that allows maintaining cell viability at a level of approximately 70– 80% [138, 139]. However, it is quite difficult in extrusion bioprinting to get an equivalent with a high cellular density and a clear distribution of spheroids, which results in uneven maturation of equivalents. Moreover, extrusion bioprinting has a rather low resolution and is not suitable for cases where it is necessary to place a spheroid in a clearly specified position when reproducing complex tissue architecture.

Aspiration and laser bioprinting have the highest resolution. Aspiration bioprinting allows placing a spheroid with an error of maximum of 11–15% relative to its size [98]. High accuracy allows placing spheroids close to each other and clearly controlling the distance between them, as well as the mechanism of equivalent maturation (fusion, migration, or processes formation). However, this is a very time-consuming method, it takes approximately 20 s to move one spheroid [159]. The spheroids are moved by aspiration force, which must be carefully chosen depending on the mechanical properties of the object. It is better to use more mature spheroids with a high content of extracellular matrix to reduce deformation. However, the maturation of the equivalent in such a case takes longer time, as the spheroids fuse worse [98, 106, 108]. Laser bioprinting allows positioning the spheroids with an accuracy of 1 μm as well as ensures high cell viability [148, 149]. This method is mainly used for bioprinting with cells, and the spheroids were used in only a few studies.

The Kenzan method also has a high resolution (up to 1 μm) and allows placing the spheroids in close proximity to each other, which results in formation of dense microtissue. However, it limits the size of spheroids and cannot be used with objects with a diameter of less than 400 μm. At that, the optimal size of the majority of spheroids was established to be 200–300 μm. Otherwise, the viability of cells in the central spheroid area decreases. Moreover, the Kenzan method does not allow creating bioequivalents of complex shapes and is mainly applicable for printing simple flat or tubular structures [136, 158].

Thus, when choosing a bioprinting method, one shall pay attention to such critical parameters as the phenotype of the cells included in the spheroids, their mechanical properties, the desired cellular density of the resulting tissue equivalent, and the degree of spheroid differentiation. To print dense, elaborated bioequivalents, one shall better give preference to aspiration and laser bioprinting, as these methods allow positioning spheroids with high accuracy and in close contact. If it is necessary to print large-sized equivalents, one shall better use extrusion bioprinting, as this is a simplest and fastest method.

It is doubtless that, when using the bioprinting method, one shall consider many peculiarities, and, unfortunately, there are no clear rules, which would guarantee the receipt of the required tissue equivalent. However, bioprinting using spheroids is one of the most promising methods in tissue engineering. In the nearest future, the method will definitely be optimized for creation of bioequivalents of various types, which will open up opportunities for both replacement therapy and development of adequate models for testing the effectiveness and safety of medications.
